# Exploring the Nucleophilic *N*- and *S*-Glycosylation Capacity of *Bacillus licheniformis* YjiC Enzyme

**DOI:** 10.4014/jmb.2001.01024

**Published:** 2020-03-20

**Authors:** Puspalata Bashyal, Samir Bahadur Thapa, Tae-Su Kim, Ramesh Prasad Pandey, Jae Kyung Sohng

**Affiliations:** 1 Department of Life Science and Biochemical Engineering, Sun Moon University, Asan 31460, Republic of Korea; 2 Department of Department of Pharmaceutical Engineering and Biotechnology, Sun Moon University, Asan 31460, Republic of Korea

**Keywords:** *Bacillus licheniformis*, natural product, glycosylation, diversification

## Abstract

YjiC, a glycosyltransferase from *Bacillus licheniformis*, is a well-known versatile enzyme for glycosylation of diverse substrates. Although a number of *O*-glycosylated products have been produced using YjiC, no report has been updated for nucleophilic *N-*, *S-*, and *C-* glycosylation. Here, we report the additional functional capacity of YjiC for nucleophilic *N-* and *S-* glycosylation using a broad substrate spectrum including UDP-α-D-glucose, UDP-*N*-acetyl glucosamine, UDP-*N*-acetyl- galactosamine, UDP-α-D-glucuronic acid, TDP-α-L-rhamnose, TDP-α-D-viosamine, and GDP-α-L- fucose as donor and various amine and thiol groups containing natural products as acceptor substrates. The results revealed YjiC as a promiscuous enzyme for conjugating diverse sugars at amine and thiol functional groups of small molecules applicable for generating glycofunctionalized chemical diversity libraries. The glycosylated products were analyzed using HPLC and LC/MS and compared with previous reports.

Glycosylation is a well-characterized glycosyltransferase (GT) enzyme-catalyzed reaction, in cells, involved in metabolism, cell integrity, molecular recognition and pathogenicity, and post-modification of secondary metabolites during biosynthesis [1–4]. GTs are ubiquitous in nature and transfer sugar moieties from activated nucleotide diphosphate sugars (NDP-D/L-sugars) to acceptor molecules. Leloir GTs are NDP-sugar dependent and transfer sugar units to lipid, nucleic acid, natural products, and other small molecules at nucleophilic oxygen (*O*-), nitrogen (*N*-), sulfur (*S*-), or carbon (*C*-). According to the recent CAZy classification (http://www.cazy.org/), GTs are classified into 110 different families. Among them, GT1 family proteins are inverting enzymes having GT- B type 3D structure transferring diverse sugars to small molecules [5, 6]. The glycosylation of natural products (NPs) influences the physical, chemical and biological properties of the parent molecules. Especially, the sugar conjugation to therapeutically important NPs alters the pharmacological and pharmacokinetic properties including water solubility, stability, specificity, as well as biological actions of the compounds [7, 8].

Due to the emerging resistance to the different therapeutics, recent research has been focused on designing/ developing bioactive molecules by modification of previously known compounds using various approaches such as by applying microbial enzymes and cells as biocatalysts. Glycosylation is one of the most prominent tools to create glycoside libraries of bioactive small molecules as glycosylation results in an alteration in the pharmacokinetic properties of the parent compounds [9]. In this context, the search for novel glycosyltransferases with tolerance to diverse sets of donor substrates and acceptor compounds is expanding in importance. GTs from various organisms have been used to glucosylate diverse sets of plant natural products, specifically flavonoids [10, 11].

In this study, we have investigated the application of a GT, YjiC from non-pathogenic *Bacillus licheniformis* DSM 13 strain for glycosylation of various industrially important amino (NH_2_) and thiol (SH) functional group- containing acceptor substrates. YjiC has been extensively studied for its donor and acceptor substrate promiscuity towards nucleophilic *O*-glycosylation of diverse sets of natural products using NDP-D/L-sugars as donor substrates [12, 13]. Nucleophilic *N*-, *S*-, *C*-glycosylation is regarded as rare in comparison to *O*-glycosylation of natural products. This puts the emphasis on research with those GTs capable of not only *O*-glycosylation but also able to generate other natural products with uncommon glycosidic linkages. GTs able to form C-C glycosydic linkages are gaining attention because of the stability of C-C bonds, and the resulting activity of both glycosyl and aglycone parts [14]. Likewise, *N*- and *S*-linked glycosidic linkages are also equally important for developing novel natural products with potential biological activity. In this study, we explored promiscuous YjiC enzyme for *N*- and *S*-glycosylation of different thiol and amino group-containing compounds as acceptor substrates and various NDP-D/L-sugars as donor substrates.

Previously constructed pET28-YjiC [15, 16] was used to produce enzyme in *E. coli* BL21(DE3). The His6-fused YjiC enzyme purified using Ni++ affinity chromatography (Fig. S1) was used for in vitro enzyme reactions. The *O*-, *N*- and *S*- trifunctional property of pure YjiC was investigated by reacting the enzyme with 2 mM of 3-dichlorophenol (**1**), 3-dichloroaniline (**2**) and 3-dichlorobenzenethiol (**3**), 4 mM UDP-α-D-glucose (UDP-Glu), in 100 mM Tris-HCl buffer containing 10 mM MgCl2 at 37oC for 3 h (Fig. 1). Reactions were analyzed using high-performance liquid chromatogram (HPLC) which showed the conversion of **1** (retention time (*t*_R_ 14.5 min) to **1a** (*t*_R_ 10.7 min) (Fig. 2Ai-2Aiv), conversion of **2** (retention time (*t*_R_ 13.6 min) to **2a** (*t*_R_ 10.5 min) (Fig. 2Bi-2Biv), and **3** (retention time (*t*_R_ 17.9 min) to **3a** (tR 11.3 min) (Fig. 2Ci-2Civ). Analyzing all three samples by high-resolution quadrupole time-of-flight electrospray ionization mass spectrometry (HR-QTOF ESI/MS) in positive ion mode showed the masses of *m/z*^+^ 347.0056 Da, *m/z*^+^ 324.0398 Da, and *m/z*^+^362.9830 Da, which exactly resembled the glucose- conjugated mass of **1**, **2** and **3** respectively (Fig. 2). After analyzing the conversion rate of these newly derived products (**1a**, **2a,** and **3a**), interestingly, conversion of **3a** was maximum, which was 43.8% compared to **1a** (11.3%) and **2a** (25.2%) (Fig. 3B). We have compared these products (**1a**, **2a**, and **3a**) with those generated after glycosylation of **1**, **2**, and **3** using YdhE and YojK [17]. Since the HPLC chromatogram, UV-VIS and ESI/MS spectra of products produced by YjiC aligned exactly with the products of YdhE and YojK (data not shown), we confirmed conjugation of glucose in β-configuration.

Furthermore, the substrates **1**, **2** and **3** were reacted with eight different types of NDP-D/L-sugar donors including UDP-α-D-galactose (UDP-Gal), UDP-α-D-glucuronic acid (UDP-GlcA), UDP-α-D-*N*-acetyl- galactosamine (UDP-GalNAc), UDP-α-D-*N*-acetyl-glucosamine (UDP-GluNAc), TDP-α-D-2-deoxyglucose (TDP-2dGlc), TDP-α-D-viosamine (TDP-vio), and NDP-L-sugars (TDP-α-L-rhamnose (TDP-Rhm), and GDP-α-L-fucose (GDP-fuc)) (Figs. 3C, S2-25). When the reaction was carried out using **1** as an acceptor substrate and with eight different sugar donors, HPLC-PDA and HR-QTOFESI/MS analysis confirmed only two products, **1e** and **1h** (Figs. S5 and S8). No product was detected with other NDP-sugar donors (Figs. S2-S7 and S9). When identical reactions were carried out with **2**, distinct conversion of **2** was seen to **2b**, **2e**, and **2h (**Figs. S10-S16**)** while with the rest of the other sugar donors, products (**2c**, **2d**, **2f**, **2g**, **2i**) were detected in ESI/MS (Figs. S11-S17). Similarly, in the identical reactions with **3**, the glycosylated products were produced with all eight sugar donors (Figs. 3B and S18-S25). These results revealed that YjiC enzyme was also able to form diverse sugar-conjugated *N*- and *S*-glycosidic linkages. Interestingly, the conversion to glycosides was higher towards the thiol functional group rather than the amine and hydroxyl functional groups (Fig. 3B).

After exploring the potential of YjiC to tolerate diverse NDP-D/L-sugars and transfer them to amine and thiol functional groups, we carried out enzyme reactions with purified YjiC with a set of aglycone compounds containing amine and thiol functional groups (Fig. 4) under the same set of reaction conditions using UDP-Glu as sugar donor. The HPLC-PDA and HR-QTOF ESI/MS analysis revealed glycosylated products of the substrates 4, 6, 7, 8, 10, 11, 12, 14, 16, 17, 19, and 20 belonging to the different classes of natural products (Figs. S26-S37). However, with the substrates 5, 9, 13, 15, and 18, the products were not detected as expected (Figs. S38-S41).

Since conjugation of bulky sugar groups to natural products dramatically changes polarity, water solubility, and binding affinity of parent compounds, glycosylation has been developed as a tool to engineer natural products with different sugars [17]. Importantly, conjugation of different sugars in the same parent molecule helps to study the effect of glycodiversification in altering biological activities, which eventually helps in studying the structure activity relationship of newly developed compounds to various targets. Additionally, glycans are directly involved in cell-cell interactions, binding to molecules, and recognition in cells. Many microbial natural product glycosides contain deoxysugars and are known to play crucial roles in biological activity, stability and binding affinity of drugs to target sites [18]. Thus, conjugation of sugars to drug scaffolds helps to gain new or enhanced binding affinity. Likewise, conjugation with amino deoxysugars to natural products changes the basicity of the molecules [19] while thiol-linked and amine-linked glycosides also bring changes in physical, chemical, and biological properties [20].

In summary, different experimental results demonstrated that YjiC from *B. licheniformis* has very versatile, functional potential to transfer diverse sugars to not only nucleophilic *O*- but also towards *N*- and *S*- groups, thus expanding possible use of YjiC for the chemical library of *N*- and *S*-glycosides. Additionally, the enzyme has more activity with thiol glycosylation in comparison to hydroxyl and amine group-containing compounds. Cumulatively, YjiC could serve as a potential biocatalyst for the glycodiversification of various drug scaffolds, like YdhE and YojK [21].

## Figures and Tables

**Fig. 1:**
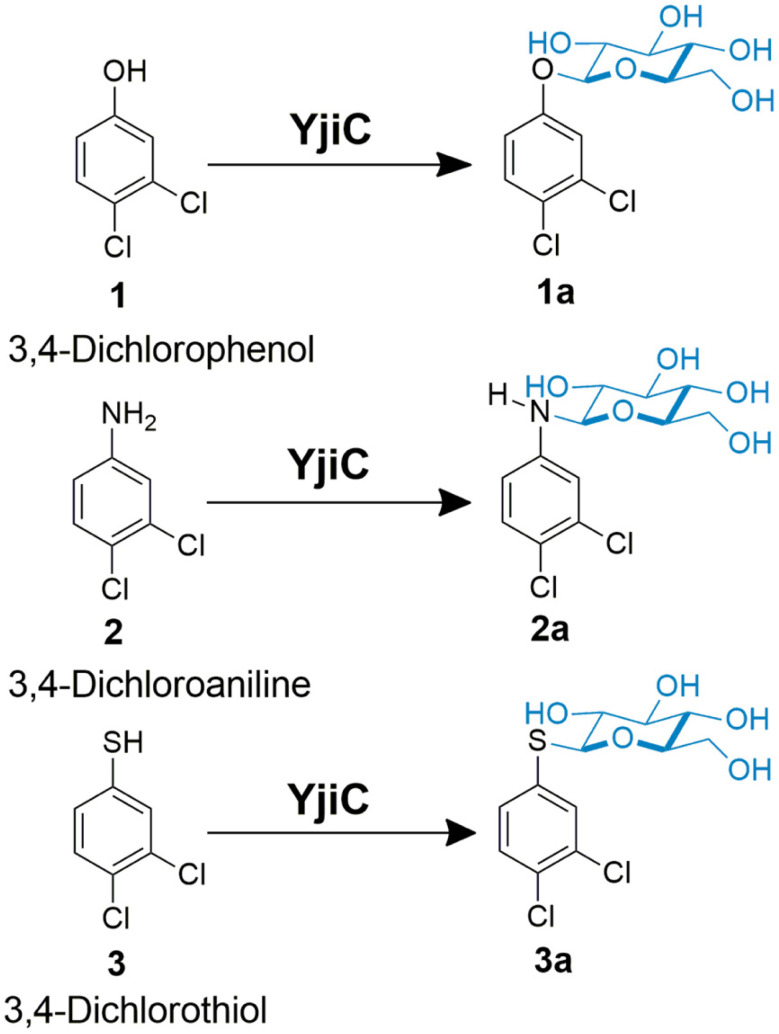
*O*, *N*, and *S*-glycosylation reactions catalyzed by YjiC. Reaction ingredients were as follows: 4 mM UDP-Glu 2 mM of aglycon, 10 mM MgCl2, 100 mM Tris-HCl (pH 8.0), 37 °C/3 h, and YjiC enzyme.

**Fig. 2:**
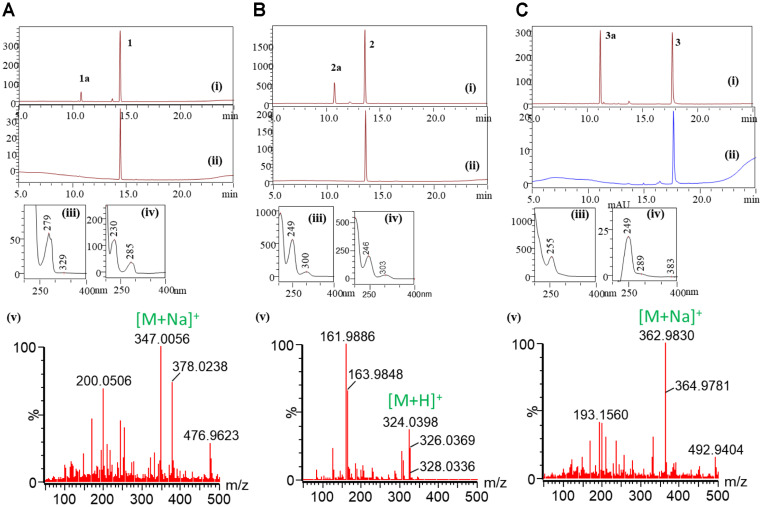
HPLC-PDA and HR-QTOF ESI/MS analysis. (**A**) 3,4-dichlorophenol (1) (ii) and UDP-α-D-glucose reaction with YjiC (i). UV/VIS analysis of product **1a** (iii) and standard **1** (iv). ESI/MS analysis of the product, **1a** in positive mode (v). (**B**) 3,4-Dichloroaniline (**2**) (ii) and UDP-α-D-glucose reaction with YjiC. (i) UV/VIS analysis of product **2a** (iii) standard **2** (iv). ESI/MS analysis of the product, **2a** in positive mode (v). (**C**) 3,4-Dichlorobenzenethiol (**3**) (ii) and UDP-α-D-glucose reaction with YjiC. (i) UV/VIS analysis of product **3a** (iii) and standard 3 (iv). ESI/MS analysis of the product **3a** in positive mode (v).

**Fig. 3:**
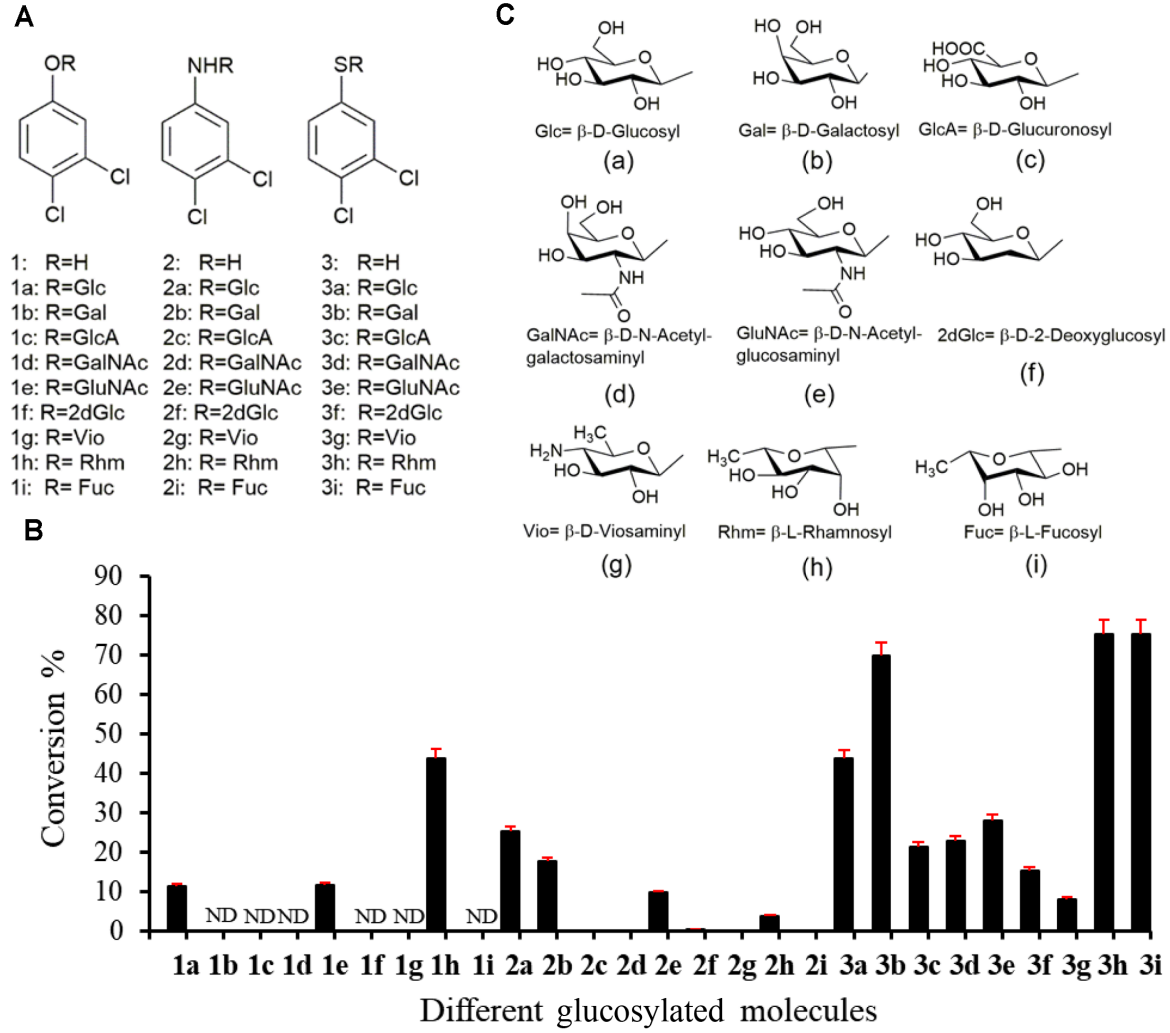
Catalytic promiscuity of YjiC at OH, NH_2_, and SH functional group-containing compounds with diverse NDP-D/L-sugars. Structures of the *O*-, *N*-, and *S*- glycosides generated. (**B**) Conversion percentage of each glycosylated molecules by YjiC. (C) Structures of different sugar moieties conjugated to compounds **1**, **2**, and **3**. ND: not detected.

**Fig. 4:**
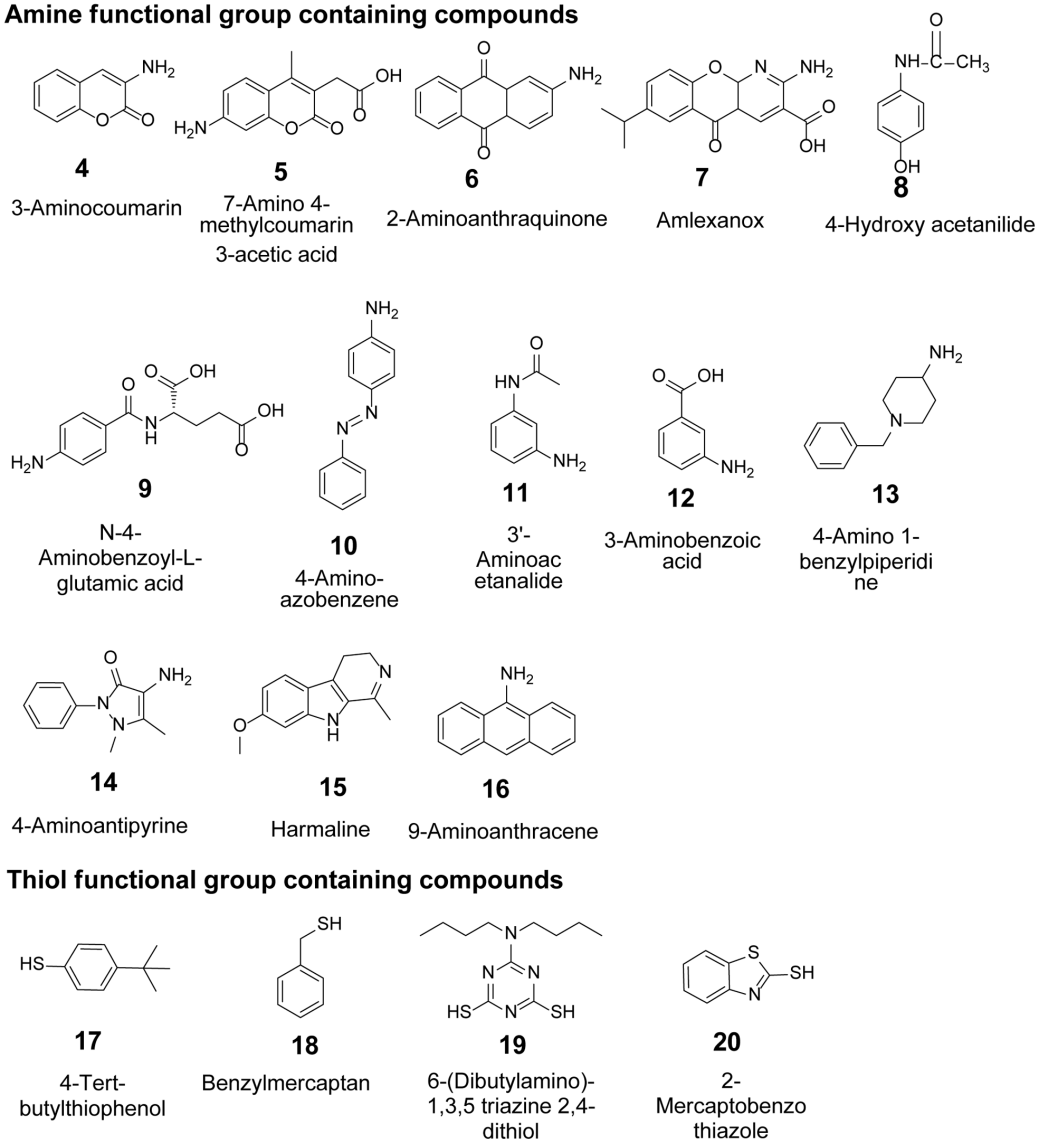
Amine and thiol functional group-containing compounds used as acceptor substrates in this study.
